# A Rare Complication of Actinomyces Abdominal Infection: A Case Report

**DOI:** 10.7759/cureus.36524

**Published:** 2023-03-22

**Authors:** Lígia Freire, Cláudia Santos, Frederico Duarte, Catarina Tavares, Catarina Q Silva

**Affiliations:** 1 General Surgery, Hospital Pedro Hispano, Matosinhos, PRT; 2 Infectious Disease, Hospital Pedro Hispano, Matosinhos, PRT; 3 Radiology, Hospital Pedro Hispano, Matosinhos, PRT

**Keywords:** abdominal actinomycosis, colovaginal fistula, infectious disease, general surgery, histerectomy

## Abstract

Intraabdominal infection by *Actinomyces* species, although a rare condition, usually occurs after a disruption of the mucosal barrier in a peritoneal organ. This infection is characterized by the development of an extended and persistent inflammatory and fibrotic reaction that can be mistaken for other pathogens or different etiologies, like tumors or inflammatory diseases. It can present as an abscess, a stricturing tissue with multiple adhesions, and/or a fistulization. Early diagnosis, targeted and prolonged antimicrobial therapy, and optimal drainage when indicated, are the key to success. The authors present a case where laparotomic hysterectomy was complicated by a superficial and an organ/space surgical site infection due to *Actinomyces* with a posterior developing of a colo-vaginal fistula that was treated surgically.

## Introduction

*Actinomyces *speciesare gram-positive bacteria, absolute or facultative anaerobes. The bacteria most frequently responsible for human infection is *Actinomyces israelli*, but others less common ones such as *Actinomyces meyeri* and *Actinomyces*​​​​​​​* odontolyticus* are also described as agents responsible for this condition [1,2]. Hematogenous dissemination is extremely rare [1,3]. These bacteria belong to the human commensal flora, present in the oropharynx, gastrointestinal, and urogenital tract but could be pathogenic when invading necrotic tissue or in case of mucosal barrier disruption [2].

Actinomycosis is a rare inflammatory disease, characterized by a local abscess formation in an initial stage, followed by a chronic and extended dense fibrotic reaction that can lead to stricturing, abundant granulation of local tissue (mimetizing a mass formation like a malignant process or an inflammatory pathology), or fistula formation (internally or cutaneously) [1,2]. 

Human *Actinomyces *infection is more common in the cervicofacial region (>50%) and is related to poor oral hygiene and dentition or as a complication of dental extraction [1,4,5]. Around 15% to 20% of the infection is thoracic, and abdominal *Actinomyces* account for 20% of cases [1,6]. Some risk factors related to abdominal actinomycosis are surgery, trauma, tumors, or a perforated viscus [7]. The most common causes are a history of appendicitis/diverticulitis with perforation or the use of intrauterine contraceptive devices [2]. There is no correlation between the disease and the social environment. The infection is more prevalent between adolescence and young adults which overlaps the age of its commonest etiology, appendicitis [1,8]. Diagnosis is made with bacteriological identification of *Actinomyces* from a sterile site by surgical biopsy or pus [2].

## Case presentation

We present the case of a 42-years-old Caucasian woman with a history of smoking, alcohol consumption, ex-drug addiction (non-intravenous), an ectopic pregnancy rupture, a surgically drained perianal abscess, and a fistulectomy due to a perianal fistula. Five days before the current hospital admission, the patient underwent a total hysterectomy in the context of uterine myomatous disease with anemia. She was on iron supplementation and hemoglobin at the time of the surgery was 10g/dL, without the need for a blood transfusion. No complications or difficulties were described during the surgery. The patient presented at the emergency room with lower abdominal pain and surgical wound purulent discharge with evidence of a pelvic abscess near the vagina on a CT scan. She was admitted for transvaginal drainage and empiric antibiotic therapy was started.

**Figure 1:**
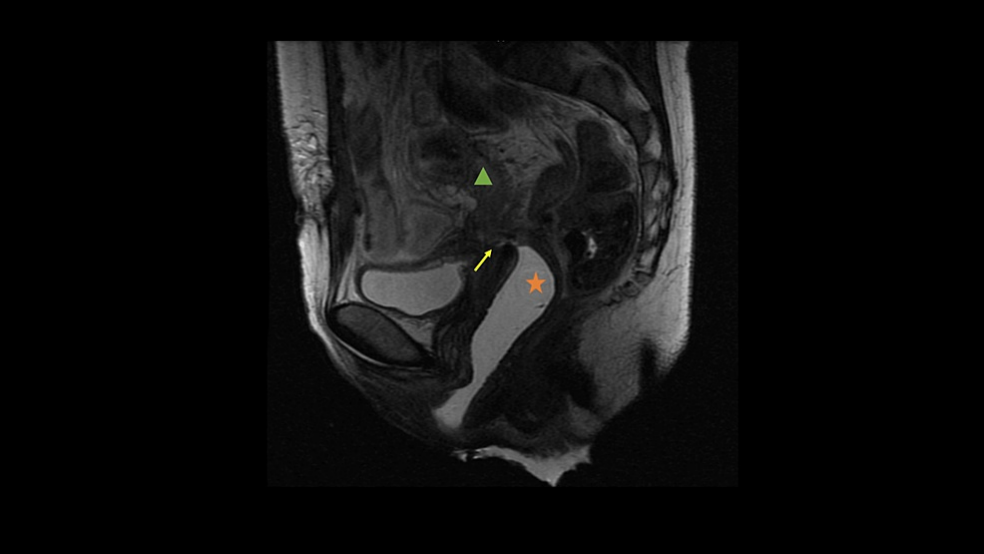
MRI showing a fistula between the sigmoid colon and vagina Green triangle: Sigmoid colon, Orange star: Vagina, Yellow arrow: Fistula

Microbiology from the wound revealed an *A. ondontolyticus* and a *Propionibacterium granulosum* (both amoxicillin/clavulanic acid sensitive). Two weeks later, due to pelvic abscess persistence on the CT scan, she underwent drainage by laparotomy, resulting in a small iatrogenic laceration of the sigmoid colon serosa that was repaired by the general surgery team with a simple serous suture. New samples of intra-abdominal purulent content were sent for study, allowing the identification of *A. meyeri*, *Prevotella intermedia*, *Peptostreptococcus, *and *Streptococcus agalactiae,* all with the same sensitivity profile. An infectious disease specialist reviewed the case, and antibiotic therapy with amoxicillin/clavulanic acid was initiated. Fifteen days later, the patient developed a colovaginal fistula, with vaginal fecal discharge. The fistula was proved with the instillation of methylene blue in the rectum that extravasated through the vagina. To better characterize the fistula, a rectosigmoidoscopy was performed but it was inconclusive due to adhesions. The MRI showed retraction areas between the sigmoid colon, small bowel, vagina, and bladder and a fistula between the sigmoid colon and vagina (Figure 1). A terminal colostomy and a mucous fistula of descending colon were performed to control the fistula. 

The patient was discharged 15 days after the last surgery. She completed two months of intravenous amoxicillin/clavulanic acid (2.2g every eight hours) plus 10 months of oral amoxicillin after that. No recurrence was observed. One year later the patient was admitted for a sigmoidectomy and intestinal reconstruction with colorectal anastomosis.

## Discussion

Abdominal *Actinomyces* infection can have many causes, sometimes unknown, and is associated with some risk factors such as previous abdominal surgeries, as seen in our case. When present, it is usually associated with a prolonged and intensive desmoplastic reaction that leads to extensive adhesions that can progress to fistula formation between the abdominal organs. Because of this behavior, it can be mistaken for tumors or inflammatory diseases.

A clear diagnosis is crucial to direct therapeutic strategy as early as possible. Tissue from surgical biopsy or pus should be collected for bacteriological identification of A*ctinomyces*. The growth of *Actinomyces* is slow; it appears within at least five days and may take up to 15 to 20 days. Thus, at least 10 days of incubation are required before the conclusion of a negative culture [2]. Prolonged antibiotic therapy (six to 12 months) is essential since the intense desmoplastic reaction associated with *Actinomyces* infection limits drug penetration. Penicillin or amoxicillin, at high doses, are the drugs of choice [1,2]. *Actinomyces* spp. doesn’t produce beta-lactamases so there is no need to combine amoxicillin with beta-lactam inhibitors unless there are others pathogens involved. In our case, co-infection with other agents was identified, and the unavailability of amoxicillin for the isolated intravenous formulation did not simplify the pharmacological plan in the hospital phase. The presence of “companion microbes” contribute to the inflammatory process inhibiting host defenses or reducing oxygen tension [2,9]. That’s why it has been suggested that *Actinomyces* are pathogenic only with the synergic action of other bacteria [1].

Surgical resection of semisolid lesions or surgical drainage of abscesses is frequently required, not only because it allows the collection of noble samples for microbiology and thus a possible faster diagnosis, but also as a way to control the infection, reduce the bacterial burden, and decrease the possibility of recurrence and complications. In this patient, the fistula formation can be consequent to several etiologies, such as previous surgical manipulation, the iatrogenic lesion of the colonic serosa, and the polymicrobial infection. However, the prolonged infection process associated with a large local inflammatory and fibrous mass makes us believe that* Actinomyces *infection had a great impact on fistula formation as this is its usual presentation. So, if the surgical drainage by laparotomy had taken place earlier, maybe we could have prevented fistulization. Although no reference was found in the literature, given that the late recurrence of the infection is a well-described reality, these patients should be followed up whenever possible (for at least 12 months).

## Conclusions

*Actinomyces *abdominal infection is a rare condition. Clinical teams must be alert to this identity and the way it usually presents and evolves when misdiagnosed or undertreated since some complications can be life-threatening. Prompt and optimal drainage of infected and necrotic tissue combined with prolonged antibiotics are the cornerstone of abdominal *Actinomyces* treatment. The management of these patients must be performed by multidisciplinary teams, including surgery, gynecology, and infectious disease specialists. Given that late recurrence is possible, these patients should be followed up whenever possible after discharge.
